# Development of a granular bioformulation of *Achromobacter xylosoxidans* AX77 16S for sustainable onion white rot management and growth enhancement

**DOI:** 10.1038/s41598-025-10036-8

**Published:** 2025-07-30

**Authors:** Aya I. ELKasaby, Khalid M. Ghoneem, Yasser M. Shabana

**Affiliations:** 1 Faculty of Agriculture, Mansoura University, Mansoura, 35516 Egypt; 2 Seed Pathology Research Department, Plant Pathology Research Institute, Agricultural Research Center, Giza, 12619 Egypt; 3 National Council for Agricultural and Food Research, Academy of Scientific Research and Technology, Cairo, 11516 Egypt

**Keywords:** Biological control, Endophytic bacteria, Microbiota, Onion, White rot, *Stromatinia cepivora*, Biological techniques, Biotechnology, Plant biotechnology

## Abstract

Onion white rot, caused by *Stromatinia cepivora*, is a major soil-borne fungal disease that severely affects onion production. This study introduces a novel endophytic bacterium, *Achromobacter xylosoxidans* AX77 16S, isolated from healthy onion seeds, as a potential biocontrol agent and growth promoter. Out of 78 bacterial isolates screened, AX77 16S showed the strongest antifungal activity, inhibiting pathogen growth by 50% in dual culture assays. Its application significantly enhanced seed germination (up to 82%) and improved shoot and root development, increasing vigor indices by over 100% compared to untreated controls. The strain was identified by biochemical and molecular techniques and registered in GenBank (ON679518). GC–MS analysis revealed ten bioactive compounds in the bacterial filtrate, while scanning electron microscopy confirmed its damaging effects on fungal structures. Additionally, AX77 16S was formulated into a granular product that remained viable for up to one year. This study presents a promising, sustainable, and cost-effective alternative to chemical fungicides for managing onion white rot and promoting crop growth.

## Introduction

The cultivation of onions (*Allium cepa* L.) extends back more than 5000 years, making them one of the earliest vegetable crops cultivated by humans. Today, Onions rank among the most widely grown vegetables worldwide^1^. It is an important crop, produced both for local consumption and export. Onions are rich in bioactive compounds, like flavonoids, which have significant medicinal properties^2^. In 2021, global onion production reached 106.59 million tonnes, harvested from 5.78 million hectares^3^. Egypt produced 3.31 million tonnes of onions from 94,457 hectares, achieving a yield of 35.0684 tonnes per hectare^3^. Onions can be attacked by a wide range of phytopathogens, including bacteria, fungus, viruses, and nematodes, leading to serious diseases and economic losses^4^. Among these, white rot disease, caused by the fungus *Stromatinia cepivora* Berk (formerly *Sclerotium cepivorum* Berk.) Whetzel poses the greatest threat and is a significant constraint on global onion production^5^. This soil-borne fungus attacks onion roots throughout the growing season, potentially resulting in total crop loss^6–8^.

*S. cepivora* is a necrotrophic ascomycete fungus responsible for causing white rot disease in a range of sensitive *Allium* plants, including leeks, onions, and garlic. Even though *S. cepivora* is not known to produce asexual spores, it survives by overwintering as sclerotia^9^. These sclerotia are small, solid, round, black structures formed by the pathogen near the end of its life cycle. They can remain dormant in the soil for years in the absence of a host and are highly resistant to extreme environmental conditions^9^. Previous reports have indicated that a variable proportion of sclerotia that naturally form on infected hosts may decompose shortly after formation for reasons that are not fully understood. However, the sclerotia that survive this decay phase can persist in the soil for up to 20 years, even without host plants present^10,11^. Although *Allium* species are the only hosts that trigger sclerotia germination, the pathogen can spread between fields through inadequate sanitation practices, including soil movement and/or contaminated water^12^.

The aforementioned characteristics of sclerotia make managing onion white rot extremely difficult and necessitate multifaceted approaches. These tactics include, but are not restricted to, sanitation techniques like soil solarization and cultural practices such as crop rotation with non-host plants^13^, biological control using sclerotia germination stimulants, composted onion waste^14^, or fungal and bacterial bioagents^15,16^. While the chemical fungicides are often considered the most effective means of controlling this disease, individual control methods do not provide appropriate level of disease management^17^. Although synthetic fungicides are efficient, their frequent and widespread usage raises environmental concerns and poses risks to humans, animals, and beneficial microbes. Furthermore, fungicides can disrupt natural biological processes and contribute to the development of resistant fungal strains^18,19^. Therefore, it’s imperative to seek affordable, environmentally friendly alternatives that are safer for people, pets, and the ecosystem.

The term"endophytic microbiome,"also referred to as"endosymbionts,"describes the diverse microbial communities that inhabit and proliferate within plant tissues, either intra- or intercellularly, for at least a portion of their life cycle, without causing noticeable effects on the environment or on consumers^20,21^. Common endophytes may be isolated from both wild and cultivated crops of either the monocotyledonous or dicotyledonous plant families. These endophytes include a range of bacteria, fungi, and actinomycetes^22^. Endophytes can enhance soil structure and have the ability to bioremediate contaminated soils. They also support their host plants through a number of mechanisms, such as the production of natural phytohormones, lytic enzymes, and other secondary metabolites that increase plant biomass and yield under abiotic and biotic stresses^23^. Despite little research, endophytic bacteria found in seeds show significant levels of bioactivity and biodiversity within their communities^23^.

In recent years, there has been growing interest in sustainable strategies to enhance onion production while minimizing environmental impact^24^. Among these, the use of endophytic microorganisms, particularly seed-borne endophytes, has shown considerable promise. *A. xylosoxidans* is an endophytic bacterium known to colonize plant tissues without causing visible harm. In this context, Dhaouadi et al.^25^ reported the antifungal activity of *A. xyloxidans*, against isolates of *Fusarium oxysporum* and *F. solani*, achieving up to 80% inhibition, and reducing wilt disease severity in melon plants by 60%**.** Furthermore, greenhouse and field studies demonstrated the efficacy of *A. xylosoxidans* (ON955872) in protecting banana plants against leaf spot disease caused by *Curvularia lunata*^26^**.** Similarly, an endophytic strain, *Achromobacter* sp. F23KW, isolated from fenugreek seeds, exhibited notable antifungal activity by producing compounds such as 2-Butanol and 3,3′-oxybis-, along with indole-3-acetic acid, which suppressed *Rhizoctonia solani* growth by 43.75% and effectively slowed the progression of root rot disease^23^**.** More recently, the antifungal and antiaflatoxin potential of *A. xylosoxidans* ZJS2-1, isolated from the peanut rhizosphere, was confirmed against *Aspergillus* *flavus*^27^**.**

The commercial use of microorganisms for biological control of plant pathogens faces a major problem i.e., the gradual loss of their vitality and effectiveness over time. Formulating these microorganisms offer several advantages, including protecting the antagonistic organism from harsh environmental conditions such as nutrient deficiencies for survival, and providing a secure environment to shield them from destruction and adverse conditions. Thus, the antagonistic organism can remain effective and active for an extended period. Furthermore, formulated products can be easily and efficiently used by farmers, enhancing the practicality of using these beneficial antagonistic organisms^28,29^.

Granular formulations, in particular, stand out from other types due to their ease of application in the field, increased stability, and heightened resilience to adverse environmental conditions. These granular bioinoculants exhibit a pelletized form with an extended shelf life. Moreover, farmers may use them effectively and conveniently, making these formulations highly practical^30–33^. The aim of the current work was to establish a straightforward process for biotransforming the endophytic *Achromobacter xylosoxidans* AX77 16S into a bioagent formula that adds value. Prior to testing the resulting product as a growth booster, bioagent, and inducer of plant resistance against *S. cepivora*, its shelf life was assessed.

## Results

### Isolation of endophytic bacteria and their antagonistic activity

**Fig. 1:**
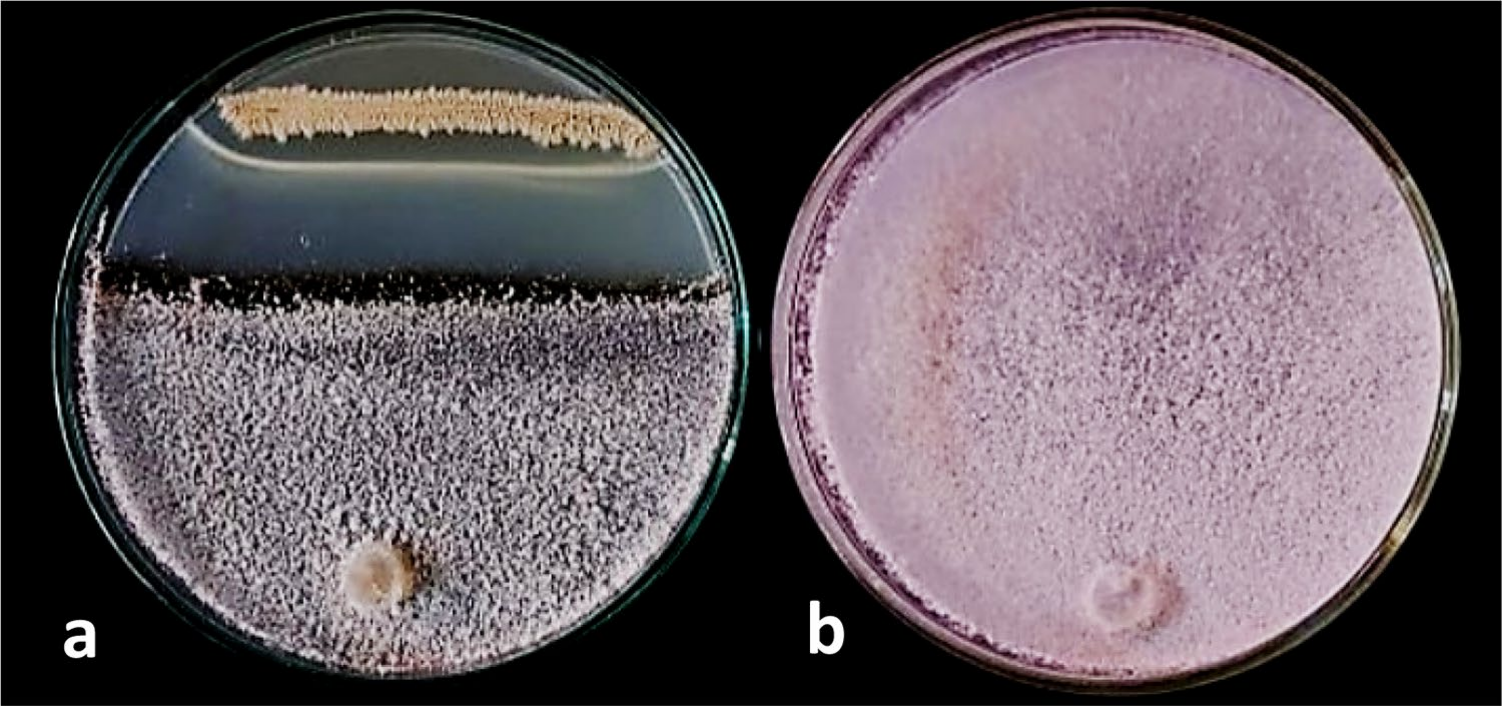
Plates demonstrating the in vitro antifungal activity of the endophytic bacterium strain AX77 16S against *S. cepivora* indicate the antagonism between the dual cultured pathogen and the bacterial strain (**a**), in compared to the growth of *S. cepivora* alone on a PDA plate (**b**).

In a dual culture test, 78 endophytic bacterial isolates from healthy onion seeds were assessed for their effects on the growth of *S. cepivora* (Supplementary Data). Most of the isolates demonstrated varying degrees of inhibition against the target pathogen. As shown in (Table S1, Fig. 1), strain No. AX77 16S exhibited the highest level of antagonistic activity, inhibiting *S. cepivora* growth by up to 50%, leading to its selection for further testing. While 61 isolates exhibited slow growth and no detectable biological activity, the remaining 16 isolates demonstrated the ability to inhibit the growth of the target pathogen. Among these, bacterial isolates NG97, NG108, NG91 and RA30 ranked next in effectiveness, reducing pathogen growth by 45.0 to 45.47%.

### Effect of endophytic A. xylosoxidans on germination

**Fig. 2:**
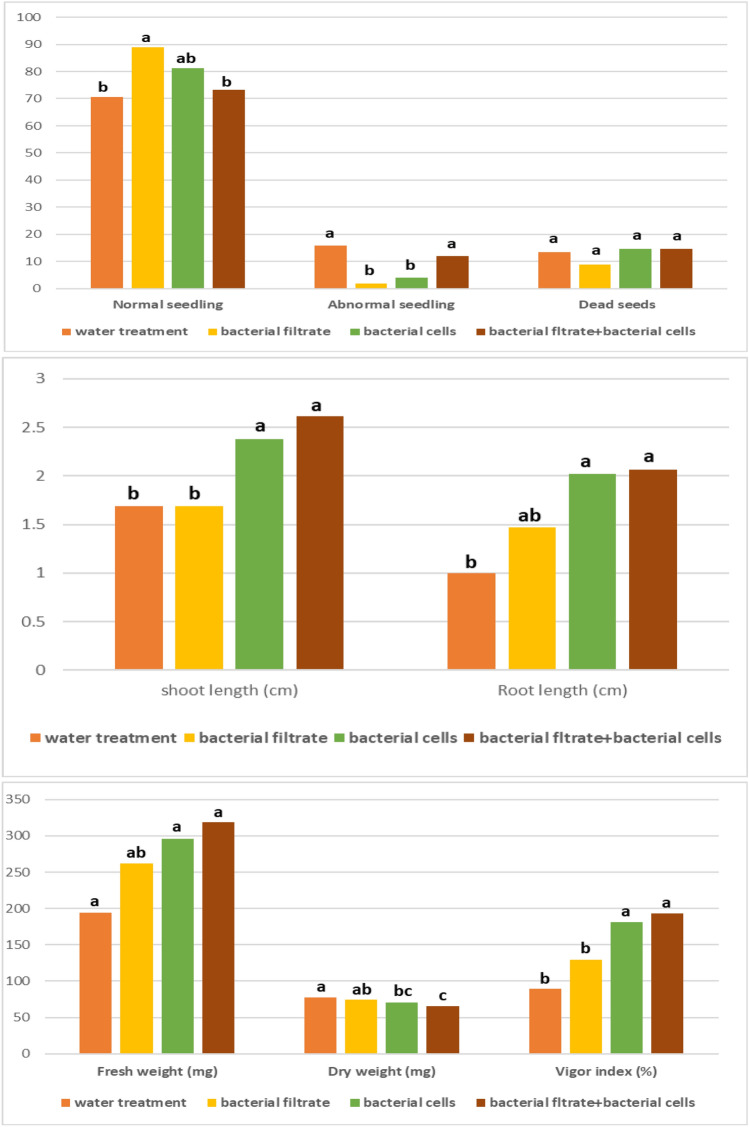
* Germination and seedling characteristics of the examined onion seeds in relation to treatment with the bacterial strain AX77 16S, n = 200 seeds/treatment. ** According to Tukey’s test, columns with the same letter (s) indicate a non-significant difference (p ≤ 0.05). *P* values for: normal seedlings = 0166, abnormal seedlings  < 0.0001, dead seeds  = 0.3449 ns, shoot length  = 0.0020, root length  = 0.0038, fresh weight  = 0.0077, dry weight  = 0.0016 and vigor index  = 0.0011.

**Fig. 3:**
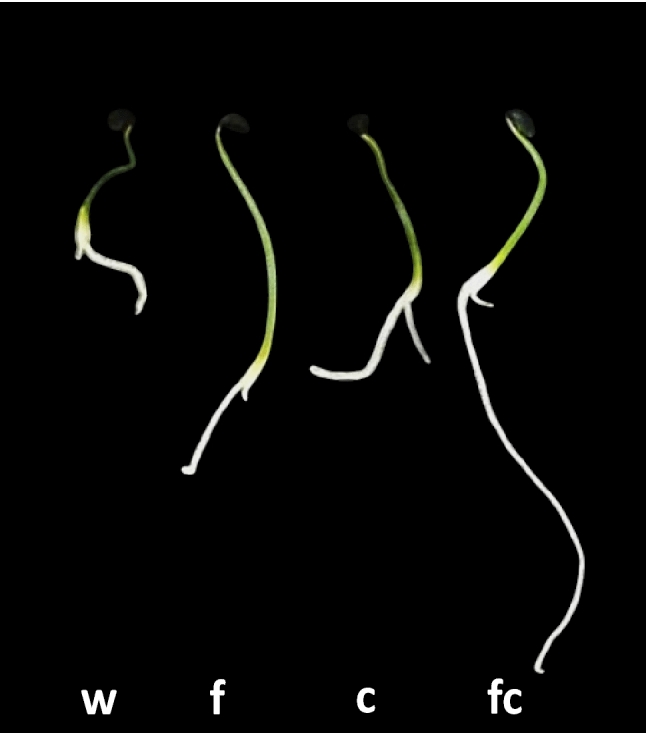
The effect of crude *A. xylosoxidans* AX77 16S CA on germination. Notes: w = water treatment; f = bacterial filtrate; c = bacterial cells: fc = bacterial filtrate + bacterial cells (100 seeds/treatment).

The effects of *A. xylosoxidans* AX77 16S derivatives (cells, filtrate, and cells + filterate) on onion seed germination and seedling traits were assessed **(**Fig. 2 and 3**)**. The germination rate for the control treatment was roughly 70.5%. In contrast, the bacterial filtrate (F) significantly increased the germination percentage to 82.0%, with no significant difference compared to its cell (C) derivative, which showed 81.25%. Additionally, both bacterial C and FC derivatives markedly improved shoot and root length compared to the control, achieving increases of 47.83, 62.11%, and 102, 107%, respectively. Similarly, the vigor index also reflected this trend, with seeds treated with C and FC derivatives exhibiting increases of 102.9 and 115.94%, respectively, compared to the control seedlings. These results indicate that derivatives of *A. xylosoxidans* can enhance onion seedling germination and vigor index, potentially reducing losses due to delayed germination.

### GC–MS profile

**Fig. 4:**
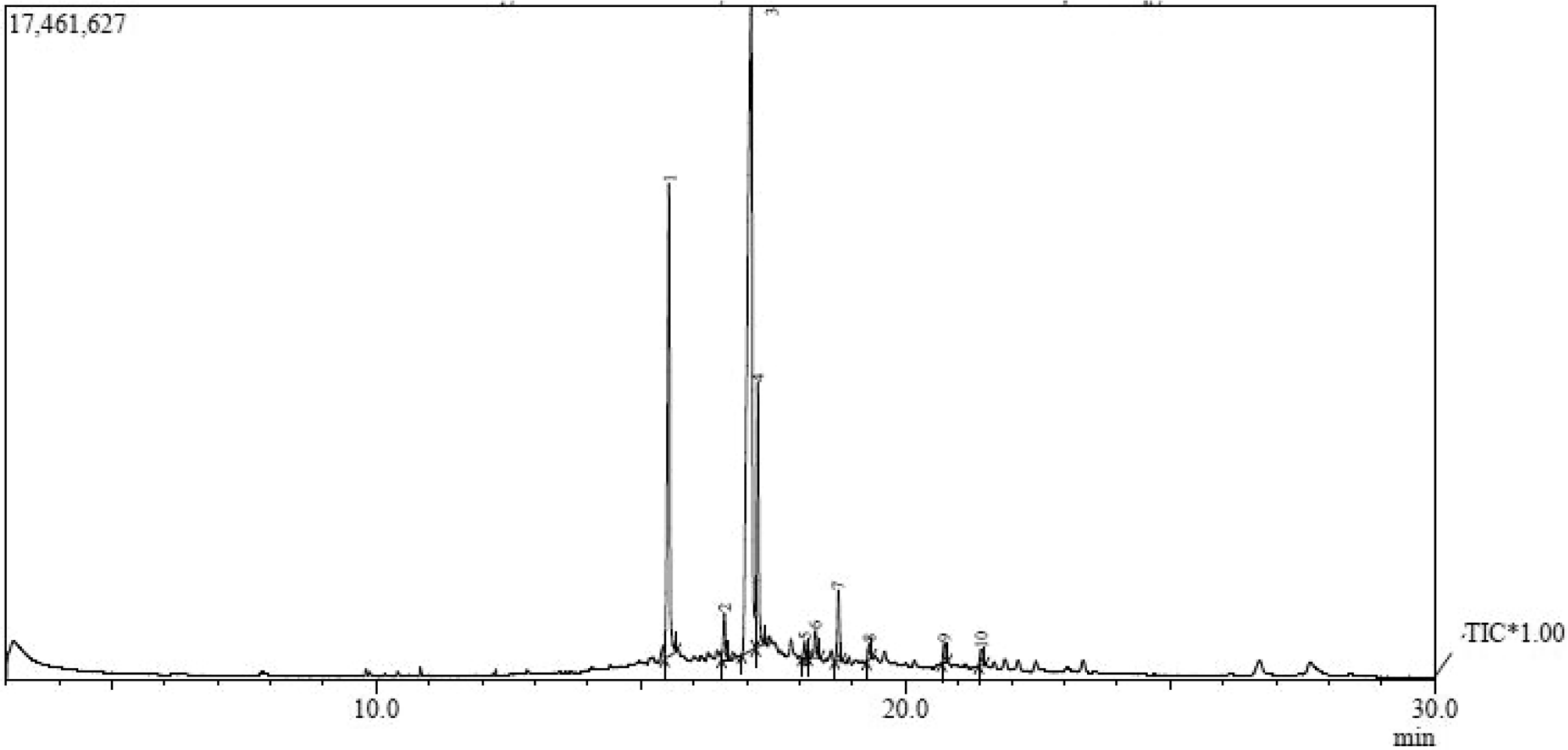
GC–MS chromatogram of bioactive compounds from AX77 16S bacterial strain. * = Total ion chromatogram of the compounds that GC–MS has tentatively identified. Arrows indicate the beginning and end of each compound peak’s base.

To better understand the bioactive metabolites, the profile of the active compounds released into the growing medium by the selected bacterial strain AX77 16S was defined using GC–MS (Table 1, Fig. 4). A total of ten major compounds were identified in the analysis. The predominant constituents included cis-vaccenic acid (58.2%), n-hexadecanoic acid (21.0%), and octadecanoic acid (10.4%). Methyl ester derivatives of 9-octadecanoic acid and cis-11-eicosenoic acid were also detected in smaller amounts, composing 1.8 and 2.8%, respectively. Additionally, five other compounds were present in trace quantities, namely, hexadecanoic acid, 2-hydroxy-1-(hydroxymethyl) ethyl ester, oleyl alcohol, cis-11-Eicosenoic acid, oleoyl chloride, and 11-Dodecen-1-ol.

**Table 1:** Quantification of chemicals identified in the hydrolysate of the bacterial strain F23KW’s supernatant using GC–MS.

Peak no	Retention time (min)	Compound	Formula	Molecular weight g/mol	Peak area	Peak area (%)	Height	Isomeric smiles
1	15.538	N-Hexadecanoic acid	C_16_H_32_O_2_	256.43	3,320,489	21.0	1,058,055	CCCCCCCCCCCCCCCC(= O)O
2	16.579	9-Octadecenoicacid, methyl ester, (E)-	C_19_H_36_O_2_	296.4879	290,213	1.8	91,676	CCCCCCCC/C = C/CCCCCCCC(= O) OC
3	17.081	cis-Vaccenic acid	C_18_H_34_O_2_	282.461	9,225,956	58.2	1,489,146	CCCCCC/C = C\CCCCCCCCCC(= O) O
4	17.215	Octadecanoic acid	C_18_H_36_O_2_	284.48	1,641,112	10.4	614,998	CCCCCCCCCCCCCCCCCC(= O) O
5	18.101	Hexadecanoic acid, 2-hydroxy-1-(hydroxymethyl)ethyl ester	C_19_H_38_O_4_	330.5	128,527	0.8	43,750	CCCCCCCCCCCCCCCC(= O) OC(CO)CO
6	18.298	Oleyl Alcohol	C_18_H_36_O	268.478	204,836	1.3	68,387	CCCCCCCC/C = C\CCCCCCCCO
7	18.735	cis-11-Eicosenoic acid, methyl ester	C_21_H_40_O_2_	324.5411	446,612	2.8	157,478	CCCCCCCC/C = C\CCCCCCCCCC(= O) OC
8	19.314	cis-11-Eicosenoic acid	C_20_H_38_O_2_	310.51	202,131	1.3	48,148	CCCCCCCC/C = C\CCCCCCCCCC(= O) O
9	20.737	Oleoyl chloride	C_18_H_33_O_10_	300.91	169,556	1.1	46,098	CCCCCCCC/C = C\CCCCCCCC(= O) Cl
10	21.436	11-Dodecen-1-ol difluoroacetate	C_14_H_24_F_2_O_2_	262.34	211,122	1.3	46,692	C = CCCCCCCCCCCOC(= O) C(F)F

### Bacterial identification

The strain AX77 of the bacterial isolate was selected for identification based on preliminary data. Morphological and biochemical analyses revealed that the bacteria are strictly aerobic, Gram-negative, straight rods that do not form spores (Table 2). The bacteria possess peritrichous flagella and are motile. The strain is capable of hydrolyzing lipids and producing indole-3-acetic acid (IAA); however, it is unable to use citrate, hydrolyze gelatin, or produce H_2_S. While it does not produce acid oxidatively from lactose, maltose, mannitol, or sucrose, it does so from xylose. The urease test yielded negative results, while tests for chitinase, xylanase, cellulase, pectinase, oxidase, catalase, nitrate reductase, indole, and citrate utilization were all positive. The identified strain is *Achromobacter xylosoxidans* AX77 16S.

**Table 2:** Biochemical characteristics of the selected endophytic bacterial strain AX77 16S.

General feature	Result	Carbohydrate fermentation	Result	Enzyme	Result	Others	Result
Gram reaction	-	D-Maltose	-	Ala-Phe-Pro-Arylamidase	-	Adonitol	-
H_2_S production	-	D-Mannitol	-	L-Pyrrolydonyl-arylamidase	-	L- Arabitol	-
Citrate utilization	-	D-Mannose	-	Beta-Galactosidase	-	Malonate	-
Gelatin hydrolysis	-	D-Sorbitol	-	Beta-N-Acetyl-Glucosaminidase	-	5-Keto-D-Gluconate	-
Lipid hydrolysis	+	D-Tagatose	-	Glutamyl arylamidase pNA	+	L- Lactate alkalinisation	-
phenylalanine deamination	-	D-Trehalose	-	Gamma-glutamyl Transferase	-	Succinate alkalinisation	-
Indol-3-acetic acid	+	D-Glucose	-	Beta-Glucosidase	-	L-Histidine assimilation	-
		D-Cellobiose	-	Beta-xylosidase	-	Coumarte	-
		Fermentation/Glucose	-	Beta-Alanine Arylamidase pNa	-	O/129 Resistance (comp.vibrio.)	-
		Palatinose	-	L-Proline Arylamidase	+	L-Malate assimilation	-
		Saccharose/Sucrose	-	Lipase	-	Ellman	-
				Tyrosine Arylamidase	-	L-Lctate assimilation	-
				Urease	-		
				Alpha-Glucosidase	+		
				Beta-N-Acetyl-Galactosminidase	-		
				Alpha–Galactosidase	-		
				Phosphatase	-		
				Glycine Arylamidase	-		
				Ornithine Decarboxylase	-		
				Lysine Decarboxylase	-		
				Decarboxylase Base	-		
				Beta-Glucoronidase	(-)		
				Glu-Gly-Arg-Arylamidase	-		

(-) Negative reaction, (+) Positive reaction.

### Molecular identification

**Fig. 5:**
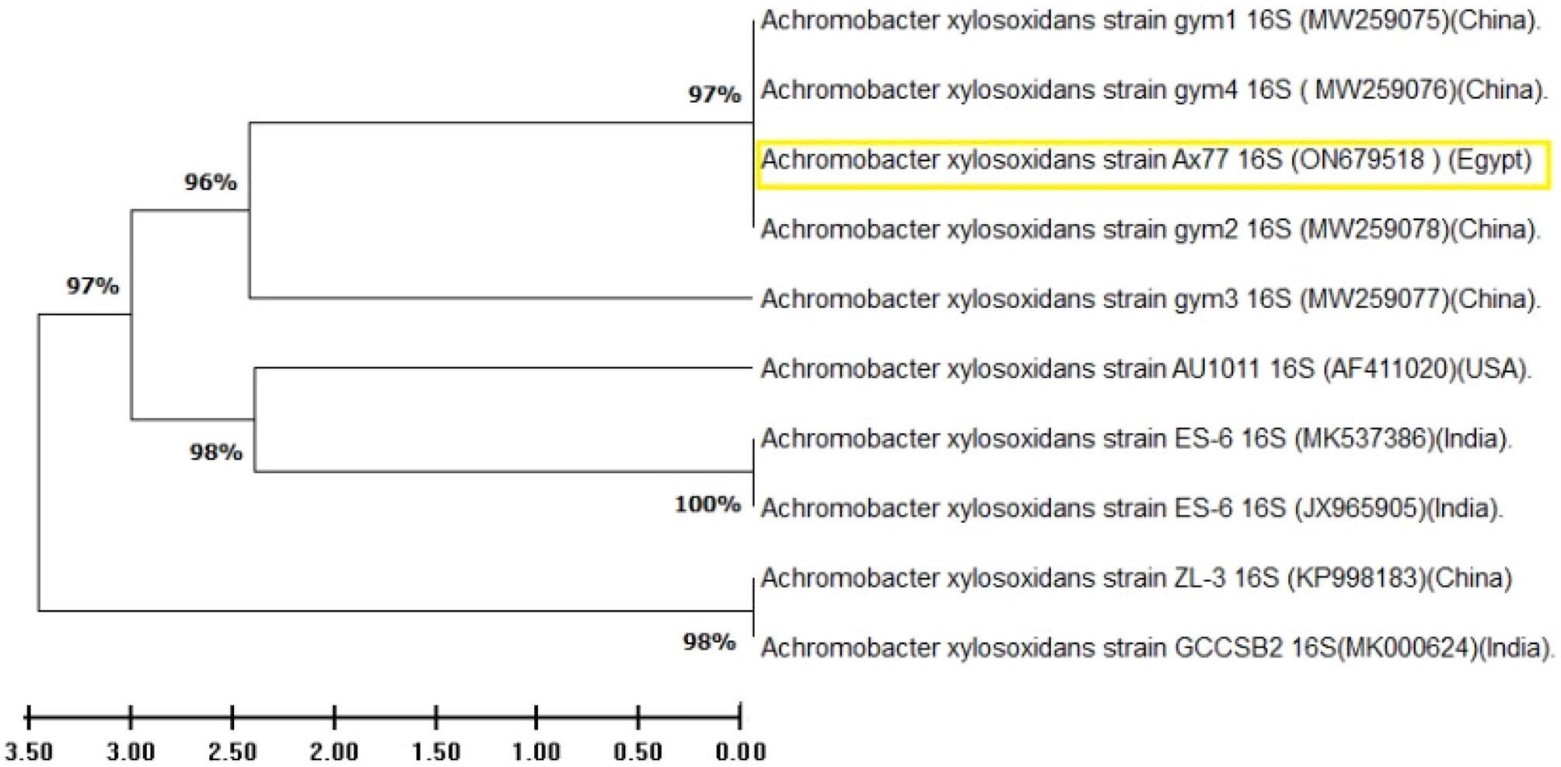
Phylogenetic tree of *Achromobacter* sp. strain AX77 generated using the neighbor-joining algorithm, illustrating its relationship with closely related species from the genus available on GeneBank.

The bacterial strain selected was molecularly characterized, confirming earlier identification. Following PCR amplification, the 16S rRNA gene sequence was examined. The AX77 16S isolate showed a significant degree of similarity to the previously identified *Achromobacter* sp. on the GenBank based on the Blast assessment (Fig. 5). The percentage of identical nucleotides across 10 sequences that clustered similar bacteria during the bootstrap test is shown below the branches. Thus, *Achromobacter* sp. strain AX77 16S was conformed as the identified strain, with its GenBank entry number was ON679518.

The BLAST tool was used to find regions of similarity between the nucleotide sequence of *A. xylosoxidans* AX77 as the query and a nucleotide database as the target (Table 3). The BLAST results indicate the high degree of similarity between the query sequence and other *A. xylosoxidans* sequences. These findings suggest a close relationship between the query sequence and various species within the genus *Achromobacter*. Furthermore, the phylogenomic analysis reveals a distinct population structure within the genus *Achromobacter*, reflecting the ecological niche and biochemical characteristics of the species. The query sequence exhibits a high percentage of identity and query coverage with most of the matched sequences, indicating strong conservation among *A. xylosoxidans* strains and related species. The E value for these hits is zero, suggesting that the matches are highly significant and unlikely to occur by chance.

**Table 3:** BLAST search results using a nucleotide sequence of *Achromobacter xylosoxidans* AX77 as the query against a nucleotide database.

Scientific name	E value	Per. ident	Accession
*Achromobacter xylosoxidans*	0	100	CP054571.1
*Achromobacter xylosoxidans*	0	100	CP053618.1
*Achromobacter xylosoxidans*	0	100	LN890477.1
*Achromobacter xylosoxidans*	0	100	CP043820.1
*Achromobacter xylosoxidans*	0	100	LS483395.1
*Achromobacter xylosoxidans*	0	100	CP158973.1
*Achromobacter xylosoxidans*	0	100	PP838568.1
*Achromobacter xylosoxidans*	0	100	PP396259.1
*Achromobacter xylosoxidans*	0	100	CP139203.1
*Achromobacter xylosoxidans*	0	100	KP998183.1

### Scanning electron microscopy (SEM)

**Fig. 6:**
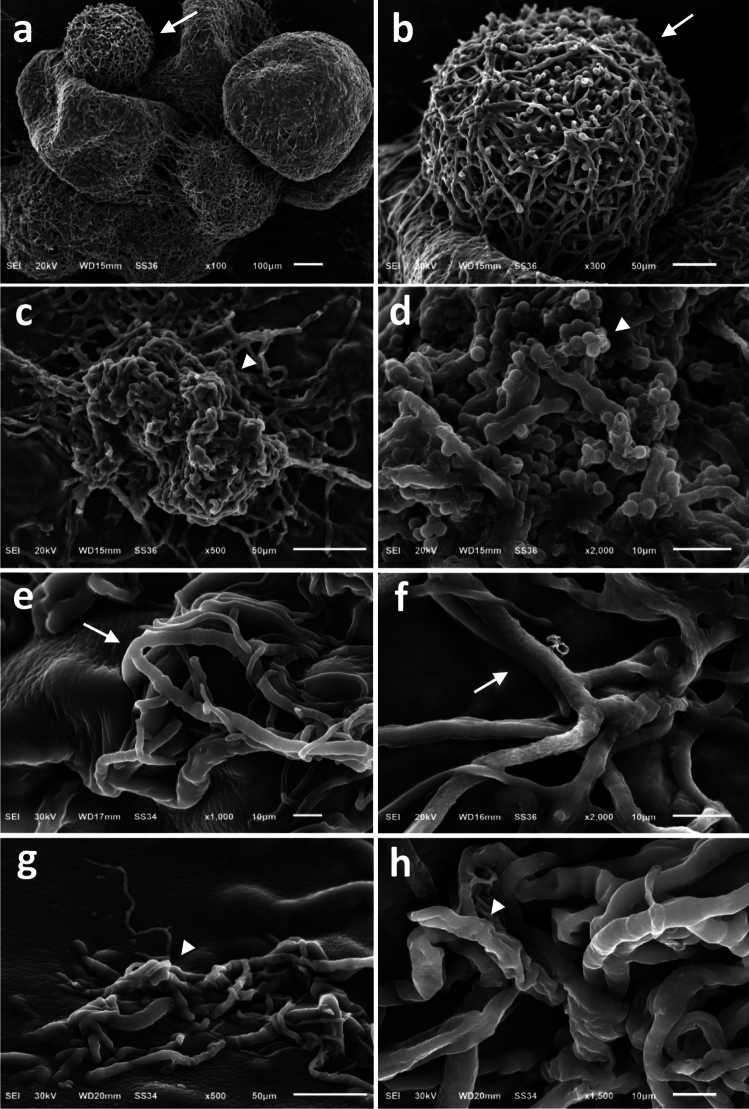
Scanning electron micrographs illustrating the antifungal effects of *A. xylosoxidans* strain Ax77 (ON679518) on the morphology of *S. cepivora* O66 in the dual culture test. Images (**a**) & (**b**) show normal untreated spherical sclerotia with intact, rough-surfaced external rind layers (arrows); (**c**), depicts treated wrinkled sclerotia with depressions and ruptured rinds; (**d**), shows small globose to subglobose rough-walled microconidia on mycelium emerging from the sclerotia (arrowheads); (**e**) & (**f**), present untreated typical well-developed branched aerial hyphae (arrows); and (**g**) & (**h**), illustrate treated twisted, curled, and collapsed hyphae (arrowheads).

SEM was used to conform the dual culture test results by examining the antagonistic effects of *A. xylosoxidans* strain Ax77 16S (ON679518) on the morphology of the fungal structures of *S. cepivora* O66 (Fig. 6). Figure 6a&b illustrates a typical untreated spherical sclerotia with intact, rough-surfaced exterior rind layers, while (Fig. 6c) depicts treated wrinkled sclerotia with surface depressions and ruptured rinds. Additionally, small globose to subglobose rough-walled microconidia can be seen on the mycelium emerging from sclerotia (Fig. 6d, arrowheads); treated samples display twisted, coiled, and collapsed hyphae (Fig. 6g&h), compared to untreated samples with well-developed branched aerial hyphae (Fig. 6e&f) (arrows).

### Estimating EGB’s shelf life

After one year of storage, granular biobactericide stored at a_w_ = 0.59 at 5 °C exhibited the longest shelf-life and highest viability for the EGB product compared to the other treatments (Table 4). The viability of all samples kept at 25 °C decreased significantly. When comparing the spore viability of treatments at a_w_ = 0.11 & 0.59 at 5 °C to the control samples, no noticeable difference was observed. Similarly, in all samples kept at 25 °C, viability dropped sharply. However, when comparing the spore viability of the two treatments at a_w_ = 0.11 and 0.59 at 5 °C to the control samples, no significant differences were found.

**Table 4:** Impact of water activity (a_w_), temperature, and duration of storage on viable colony-forming units (CFU X 10^5^) of granulated formula.

Storage (Month)	Water activity (a_w_)	EGB Viability (CFU × 10^5^)	
		**5 °C**	**25 °C**
0		755.5 a	755.5 a
1	Control	545.0 m-q	341.5 E–G
2		539.5 n-r	339.0 E–H
3		534.5 o-r	334.0 E–H
4		528.0 p-r	325.0 F-J
5		521.0 q-s	309.5 G-L
6		506.5 r-t	296.5 I-M
7		490.0 s-u	289.5 J-N
8		468.0 u-w	287.0 K-N
9		459.5 u-x	279.5 L-O
10		451.5 v-x	273.5 L-P
11		449.5 v–y	245.5 OP
12		434.5 w-z	242.5 P
1	a_w_ = 0.59	737.5 a	391.5 A-C
2		720.5 ab	379.5 B-D
3		696.0 bc	350.5 D-F
4		671.0 cd	338.5 E–H
5		662.5 c-e	332.0 E-I
6		647.5 d-g	319.0 F-K
7		621.0 f.-i	303.5 H–L
8		611.5 g-j	290.0 K-N
9		589.0 i-l	277.0 L-P
10		593.5 i-l	265.5 M-P
11		579.5 j-m	261.0 M-P
12		567.5 l-o	255.5 N-P
1	a_w_ = 0.11	660.0 c-e	471.5 t-v
2		652.5 d-f	465.5 u-w
3		649.0 d-f	458.0 u-x
4		646.0 d-g	450.5 v-x
5		632.0 e–h	444.0 v–y
6		621.0 f.-i	432.0 w-z
7		606.5 h–k	425.5 x-A
8		596.0 h–l	413.5 y-B
9		578.5 j-m	398.0 z–C
10		574.0 k-n	390.0 A-C
11		560.5 l-p	383.5 B-D
12		541.0 n-r	362.0 C-E

At the start of storage (zero time), the formulation contained  5 × 10^5^ colony-forming units. According to Tukey’s test, means followed by different letters within both column differ significantly at *P* value  ≤ 0.0.05 (n = 4). Note: when lowercase letters are exhausted, uppercase letter are used to continue the sequence.

## Discussion

A serious disease known as white rot significantly hampers the commercial cultivation of *Allium* species, leading to substantial yield losses worldwide. The ascomycetous pathogen, *S. cepivora*, produces a large quantity of infectious sclerotia that can persist in the soil for up to 20 years in the absence of a susceptible host plant^10^. Notably, this pathogen does not produce any sexual spores. Until recently, effective methods to control white rot disease have been lacking. The most widely used approach is chemical control, which relies on various agrichemicals, particularly fungicides. However, the extensive use of chemical fungicides poses several environmental and health hazards to humans, animals, and non-target organisms. Moreover, repeated applications can lead to fungicide-resistant infections. Consequently, there is an urgent need for safer, more environmentally friendly alternatives to chemical fungicides due to their inefficiency and limitations^37^. Integrating biological control into pest management programs could offer a viable strategy for combating white rot disease^34,35^.

Endophytes may provide protective benefits by producing antimicrobial compounds or by enhancing resistance through the signaling of salicylic and jasmonic acids^36^. Additional protective mechanisms include increases in lytic enzymes, expression of pathogenesis-related protein, and production of antifungal substances^37–40^. Our analysis identified substances with antibacterial activity and highlighted the release of various hydrolytic enzymes.

Both healthy and infected plantlets benefitted from the endophytes’growth-promoting effects. Although our study did not encompass the entire process of growth enhancement, it is believed that the synthesis of plant growth regulators by endophytes primarily contributes to the enhanced development of the inoculated plantlets. The two most commonly associated growth promoters linked to enhanced plant due to endophytic infection are auxins and cytokinins. Cytokinins are known to promote root elongationand increase root mass, while auxins simulate cell division, leading to large roots and faster formation of root hairs. Reports also indicate that cytokinins boost nutrient uptake and transport, contributing to overall better plant growth^22^.

In this study, a survey of bacterial endophytes associated with onion seeds was conducted. The survey yielded 123 different isolates of endophytic bacteria from onion seed samples collected from different locations, which were evaluated against *S. cepivora* using the dual culture technique. Conducting field surveys and increasing public awareness of seed health are important strategies for controlling these diseases and improving seed quality. Therefore, it is recommended that the national seed quality management program incorporate seed health testing for main crops. A recent study reported that common onion seeds often harbor serious pathogens, which can cause rot, necrosis, seed abortion, or reduce seed germination rates^41^.

Results from the dual culture tests after six days indicated that there was strong in vitro antagonistic activity of *A. xylosoxidans* strain Ax77 16S against *S. cepivora*, a finding supported by SEM observations of *S. cepivora* mycelium, sclerotia, and microconidia*.* The fungitoxic properties of *A. xylosoxidans* strain Ax77’s have been investigated by numerous researchers against a variety of soil-borne microbial pathogens, such as *Rhizopus*, *Phytophthora*, *Fusarium*, *Pythium*, *Verticillium* and *Rhizoctonia solani*^23,42^ and air-borne pathogens like *Curvularia lunata* and *Aspergillus flavus*^26,27^.

The GC–MS profile of the selected *Achromobacter* sp. AX77 16S showed the presence of highly concentrated compounds. The primary component was palmitic acid, also known as n-hexadecanoic acid, which was found in the bacterial supernatant. Ganesan et al.^43^ noted that n-hexadecanoic acid exhibits antibacterial activity against *S. aureus*, *B. subtilis*, *E. coli*, and *K. pneumoniae*. This compound may disrupt the body’s antioxidant enzyme system, potentially damaging cell structures and antifungal mechanisms against *Candida albicans* leading to the breakdown of proteins, lipids, DNA, and RNA.

The second major component identified was the methyl ester (E) of 9-octadecenoic acid, which is reported to have antibacterial effects^44^. Cis-vaccenic acid, the third main component, has been shown to exhibit antibacterial activity, a hypolipidemic impact, anticancer^45^, antihypercholesterolemic^46^, and anti-inflammatory effects^47^. The fourth principal constituent was octadecanoic acid, known for its anti-inflammatory, nematicidal, pesticidal, hemalytic, antioxidant, and 2-alpha reductase inhibitory properties against *Candida albicans*, *Aspergillus flavus*, and *A. ni*ger^48^. Octadecanoic acid has also been shown to possess a wide range of biological activities, including antioxidant, antibacterial, anticonvulsant, anti-analgesic, antiamoebic, anti-asthmatic, anti-gastric, antimalarial, anti-obesity, anti-inflammatory, anticancer, and hypocholesterolemia properties^49^. Hexadecanoic acid, also known as 2-hydroxy-1-(hydroxymethyl)ethyl ester, is the fifth main compound identified. Both hexadecanoic acid and 2-hydroxy-1-(hydroxymethyl) ethyl ester exhibit antioxidant, pesticidal, nematoidal, and hypocholesterolemia properties^50^. Additional examples of hexadecenoic acid include 2-hydroxy-1-(hydroxymethyl) ethyl ester, oleyl alcohol, cis-11-Eicosenoic acid, methyl ester, oleoyl chloride, and 11-Dodecen-1-ol difluoroacetate.

To evaluate the commercial potential of the *A. xylosoxidans* granular formulation, the shelf-life of the generated formula was assessed at two levels of storage temperature (5 and 25 °C) and two levels of water activity (0.11 and 0.59). The viability of the granular formulation was better maintained at 5 °C with a water activity of 0.59, with the possibility of extending its shelf life to a year under optimal conditions. Thus, further enhancements are needed to prolong the shelf life of the current formulation.

The bioagent’s effectiveness during storage is highly influenced by temperature; lower temperatures significantly extend EGB’s shelf life. This study determined that the ideal storage conditions for preserving propagule viability over time were 5 °C , resulting from the drastically reduced fungal metabolic activity under these conditions, compared to a higher temperature (25 °C).

*Achromobacter* spp. present a promising option for the treatment of bacterial and fungal diseases within an environmentally friendly integrated crop protection system by enhancing plant resistance to pathogens. The advantages of using *Achromobacter* in a formulated product are numerous: the biocontrol agent is protected from harsh environmental conditions, exhibits greater persistence, provides high resistance to rain and physical damage, and is supplied with nutrients to facilitate rapid activation (when exposed to irrigation water) against soil-borne pathogens like *S. cepivora*. Therefore, the formulated product is more practical, allowing farmers to utilize the bioagent with ease and efficiency^51^.

Several factors should be considered when preparing any formulation. According to various studies^31,52^, it should be easy to use and apply, effectively reach the target sites in the most appropriate form, be cost-effective to avoid burdening end users, protect the agent from adverse conditions, maintain the activity of the organism in the field, and improve the quality of soil properties. Thus, adding affordable and appropriate adjuvants, storing the inoculum at low temperatures, and maintaining low relative humidity are crucial for preserving its viability. All these safety measures were taken into account while developing the current formulation.

The current study set out to create a granular bactericide and assess its effectiveness against white rot infections caused by *S. cepivora*, as well as its impact on onion yield. Thus, the EGB was prepared for testing in field and greenhouse settings.

While *A. xylosoxidans* is widely recognized as an environmental and plant-associated bacterium, its biosafety profile warrants careful consideration before agricultural applications. Although generally non-pathogenic to healthy individuals, *A. xylosoxidans* has been identified as an opportunistic pathogen in immunocompromised patients, particularly those with underlying conditions such as cystic fibrosis, where it can cause respiratory and systemic infections^53,54^. Environmental isolates, however, often differ from clinical strains in virulence potential and antibiotic resistance profiles^55^. Recent agricultural studies have safely utilized *A. xylosoxidans* strains as biocontrol agents without adverse effects on human health or non-target organisms^56^. Nonetheless, to ensure biosafety, it is recommended that any strain intended for field application undergo comprehensive pathogenicity screening, including hemolysis testing, virulence gene profiling, and antibiotic susceptibility assessment, in accordance with established biosafety guidelines for plant-associated bacteria^57^. This precaution will help confirm the suitability of strain AX77 16S as a safe and effective bioproduct for sustainable crop management.

## Conclusions

In conclusion, this study reports a novel endophytic *A. xylosoxidans* Ax77 16S bacterial isolate with potential as a natural bioproduct for use as both a plant growth promoter and a biocontrol agent against *S. cepivora*, the causal agent of onion white rot. This strain demonstrated effective pathogen suppression and growth-promoting abilities under both in vitro and in vivo conditions. However, further research is encouraged to assess its efficacy on other plant species and across diverse environmental settings. Although *A. xylosoxidans* commonly present in the environment and occasionally associated with plants, it is typically considered non-pathogenic. Infections in humans are rare, predominantly occurring as opportunistic infections in immunocompromised individuals, such as those with cystic fibrosis. Given its plant growth-promoting traits and occasional cross-infection potential, comprehensive pathogenicity assessments are essential before considering its broader agricultural application. Overall, our findings suggest that *A. xylosoxidans* Ax77 16S represents a promising candidate for the biological control of onion white rot, with potential for future development as an agricultural bioproduct following appropriate biosafety evaluations.

## Methods

### Collection of samples

In May and June 2022, 15 samples of healthy onion seeds were collected from various governorates in upper Egypt, including Sohag, Assiut, Qena, Aswan, Luxor, Bani Suef and Minya). These locations are situated between latitudes 33°03′ N and 30°747′ N, and longitudes 29°108′ E and 22°924′ E. The samples were randomly selected in a zigzag pattern from a 50 m × 50 m area. After being gathered from fields, the onion seeds were labeled and kept at 4°C.

### Fungal contaminant

A highly pathogenic isolate of *S. cepivora* ON4 was obtained from the Onion and Garlic Diseases Department of the Plant Pathology Institute of the Agricultural Research Centre in Giza, Egypt. The pathogen was incubated at 18 °C for six days after being subculture on potato dextrose agar (PDA) plates (DIFCO, Tuker, GA, USA).

### Preparation of S. cepivora inoculum

Sorghum-soaked seeds were placed in 500 mL Erlenmeyer bottles and kept at 18 °C for fifteen days. The inoculum of *S. cepivorum* ON4 was prepared using growth plates of PDA at the same temperature. Mycelium plugs were utilized to inoculate the seeds within a week.

### Isolation of endophytic bacterial microbiome

Endophytic seed-borne bacteria were isolated using the agar plate method^58^. The seed samples were surface sterilized using three disinfectant solutions (70% ethanol for two minutes), 2% chlorine solution for one minute, and 75% ethanol for 30 s)^23^, washed with sterile distilled water for ten minutes and dried with sterile filter paper in a laminar airflow environment. Approximately twenty-five seeds were plated on a nutrient agar plate (DIFCO, Tuker, GA, USA), which was then incubated for three days under a 12-h light/dark cycle at 28 ± 2 °C with cool white fluorescent light. After two days, colonies that formed around each grain were selected, and a pure culture of the bacteria was created by spreading them over the surface.

### In vitro evaluation of endophytic bacterial isolates against Stromatinia cepivora

The dual culture plate method was employed to assess 78 bacterial endophytes isolated from onion seed samples collected from various locations against *S. cepivora*^59^. A loop of each bacterial isolate was streaked 2 cm from one edge of each PDA plate, while, a 5 mm disc taken from a 5-day culture of *S. cepivora* was placed 2 cm from the opposite edge. Control plates containing only the pathogen were also prepared. The plates were incubated at 25 °C, the experiment concluded once fungal growth fully covered the control plates. The following formula was used to assess the growth inhibition:

$$Inhibition{\text{ }}of{\text{ }}fungal{\text{ }}growth{\text{ }}\left( \% \right){\text{ }} = \;\left( {R1{\text{ }} - {\text{ }}R2} \right)/R1{\text{ }} \times {\text{ }}100$$

Where R1 denotes the linear growth of the pathogen in the control plates and R2 depicts the pathogen’s linear growth in dual culture plates.

### Biochemical profile of endophytic bacteria

#### Bacterial inoculum

To prepare the bacterial inoculum, 1 mL of bacterial suspension made by adding 5 mL of sterile tap water to bacterial slants and vortexed. This 1 mL suspension was then used to inoculate 100 mL of sterile nutrient broth adjusted to pH 7 in 500-mL Erlenmeyer flasks. After two days of incubation at 30 °C with shaking at 150 rpm, the cultured medium was centrifuged for 15 min at 4 °C and 5000 rpm. Precipitated bacterial cells resulting from centrifugation were washed using a sterile distilled water and viable bacterial counts were determined using serial dilutions and standard plate counts, ultimately adjusting to 10^8^ cfu/mL. The bacterial supernatant was stored in a refrigerator until needed.

#### Gas chromatography-mass spectrometry (GC–MS)

The supernatant from the selected bacterium was analyzed using gas chromatography coupled with mass spectrometry (GC–MS). A Shimadzu 2010 capillary gas chromatograph connected to a QP 2010 mass spectrometer (Shimadzu, Kyoto, Japan) was employed, utilizing a DB-5 ms nonpolar fused silica capillary column (30 m 0.25 mm, 0.25 µm film thickness). The oven temperature was initially set at 70 °C and increased to 200 °C, over 35 min at a rate of 3 °C per minute. The injection temperature was 200 °C, with a linear velocity of 45.1 cm/s and helium as the carrier gas at a flow rate of 1.0 mL/min. Mass spectra were acquired in electronic ionization mode (EI) at an ionization energy of 70 eV, with an ion source temperature of 200^◦^C and a solvent cut time of three minutes. The mass spectrometer scanned between 40 and 1000 m/z at 240 °C. The components of the sample were identified by comparing mass spectra and relative measurements with the WILEY and the National Institute of Standards and Technology libraries.

#### Morphological, biochemical, and molecular identification

The chosen strain AX77 16S of the isolated endophytic bacterial was examined for its morphological characteristics. After two days of growing on NA medium at 27 °C, the colony features were observed. In addition, motility and Gram staining tests were carried out. The isolate’s biological activity was determined using the VITEK-2 Compact system (bioMèrieux, Marcy-l'Étoile, France), which evaluated various factors including oxidase, catalase, urease, gelatin hydrolysis, phenylalanine, nitrate/nitrite reductase, lipid hydrolysis, indole production, H_2_S production, carbohydrate fermentation, carbon source utilization, enzyme production, growth pH range, and NaCl tolerance.

#### Molecular identification of strain Sp. AX77

The bacterial isolate AX77 16S was identified at the molecular level. Utilizing the forward (50 AGATTTGATCCTGGCTCAG 30) and reverse (50 GGTTACCTTGTTACGACTT 30) primers, the genomic DNA of the sample was extracted. This process involved ribosomal RNA analysis of 16S.The obtained 16S rRNA gene sequences were matched with the similar sequences in the GenBank database using the BLAST tool. Phylogenetic analysis was conducted using the neighbor-joining method^60^, with associated taxa grouped in a bootstrap test of 1000 replicates^61^. Unclear regions in each sequence pair were removed using pairwise deletion. Evolutionary analysis was performed using MEGA11^62^.

#### Scanning electron microscopy (SEM)

Mycelial cell samples from the contact zone were preserved for 12 h at 4 °C using a solution of 2.5% glutaraldehyde and 2% paraformaldehyde in 0.1 M sodium phosphate buffer (pH 7.4). After three washes of 15-min each with 0.1 M sodium phosphate buffer and 0.1 M sucrose, specimens were fixed in 2% osmium tetroxide buffered with sodium phosphate for 90 min. Following three additional 15-min washes in 0.1 M sodium phosphate buffer (pH 7.4), the samples were dehydrated through a graded series of alcohol (80, 90, 96, and 100%). Finally, the samples were coated with gold–palladium membranes and observed using a JEOL JSM-6510 L.V. SEM at 30 kV at EM Unit, Mansoura University, Egypt.

#### Effect of* A. xylosoxidans* on seed germination

*A. xylosoxidans* was evaluated to improve the germination and vigor index of onion seeds (Giza red cultivar), which previously had poor germination (⁓ 70%). The sample seeds were steeped separately in bio-prepared bacterial derivatives (cells, filtrate, and cells + filtrate) for two hours before being dried for one hour using sterile filter paper under a laminar airflow chamber. As a control, seeds soaked in sterile distilled water were used. Seeds were kept in a germination chamber for 14 days with 12-h light/dark cycles at a temperature of 21 ± 2 °C and 50% relative humidity. The traditional moist blotter method was used to assess the germination of 400 seeds^58^. Germinated seeds were counted at 15 and 21 days, with results expressed as germination percentages. A seed was considered germinated if its radical length measured 15 mm or greater. On day 21, the root length of 100 seeds were measured to assess their vigor. The average root length and germination percentage for each seed sample were used to calculate the seedling vigor index (SVI) using the formula^63^:

SVI = Germination percentage (%) × average root length (cm).

Bacterial filtrate, bacterial cells, and a combination of both were applied to additional cleaned seeds for 15 min. On the twenty-first day, both fresh and dry weights (mg) as well as germination percentages were recorded.

#### Production of endophytic granular biobactericides (EGB)

**Fig. 7:**
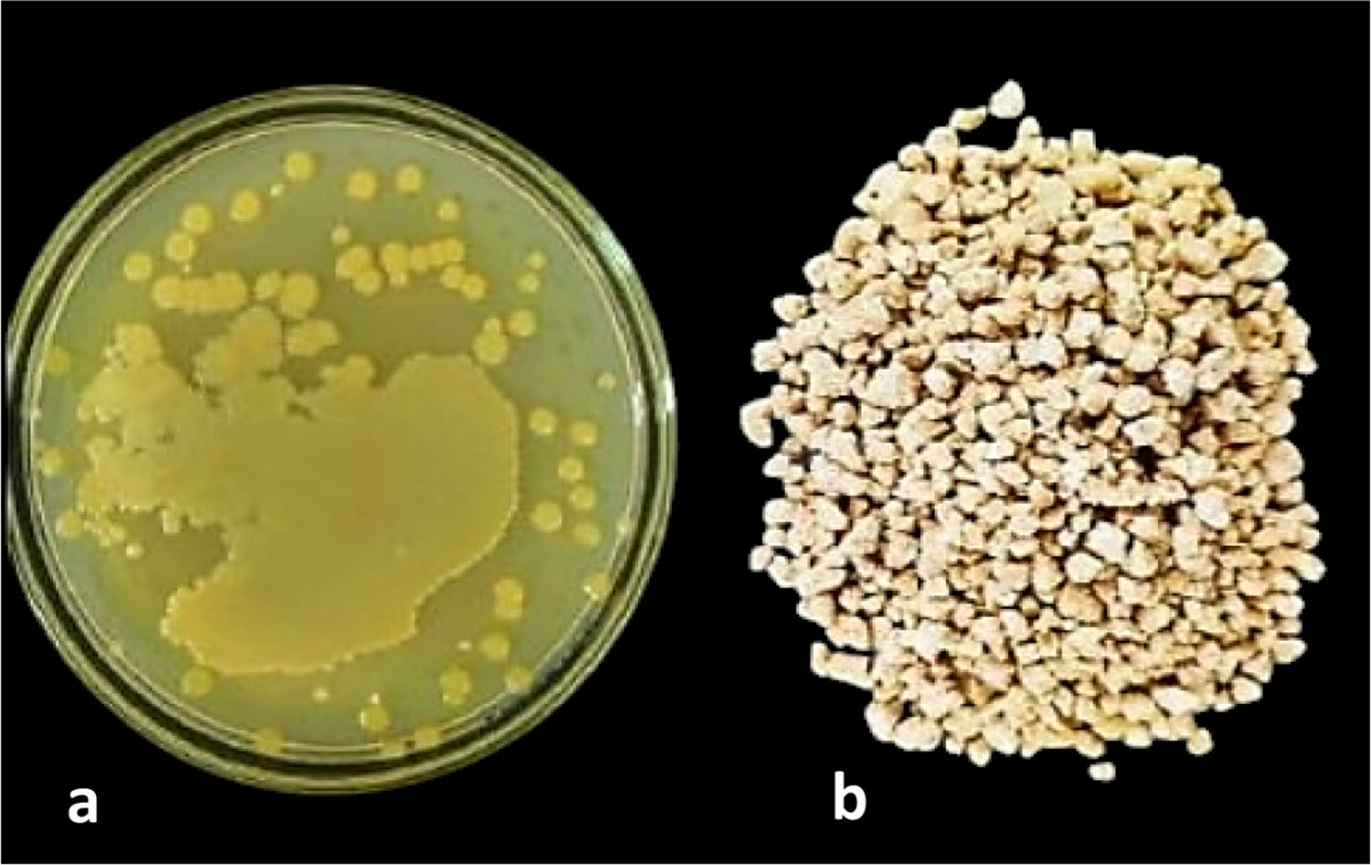
Colony growth of *Achromobacter xylosoxidans* AX77 16S on NA Plate (**a**) and its granulated formula (**b**).

The EGB dough was made by mixing 32 g of semolina, 4 g of kaolin, 6 g of yeast extract, 2 g of sucrose, 20 ml of bacterial suspension, and 3 ml of deionized water^30^. The dough was then passed through a small, manually operated pasta maker (Marcato Model Ampia 150, Padova, Italy) to create sheets, which were folded and extruded multiple times at various roller gap settings until a homogeneous mixture was achieved, resulting in sheets 1 mm thick. After drying at room temperature (25 ± 2˚C), the sheets were ground into granules in a grinder and sieved to obtain different sizes (501–2000 µm) (Fig. 7a & b). The fungal viability in the formulation, expressed as cfu/g was determined by weighing 20–60 mg of crushed or powdered granules into a tube with 10 ml of 0.15% water agar and 4 glass beads. After soaking for 15 min., the sample was vortexed until dispersed. Serial dilutions were made from this stock and plated on PDA. Fungal colonies on PDA were counted after incubation at 25 °C for 2–4 days.

#### Determination of shelf-life of granular biobacterocide

The storage stability of the EGB product was assessed at various temperatures, time intervals, and water activity levels (a_w_). This quality control test was performed shortly after manufacturing to identify any adverse effects on the EGB. The EGB was stored at 4 and 25 °C, simulating refrigerator and ambient conditions, respectively, at various a_w_ levels. To achieve consistent a_w_ level, saturated solutions (LiCl for a_w_ 0.11 at 5, and 25 °C, and Mg (NO_3_)_2._6H_2_O for a_w_ 0.59 at 5, and 25 °C) were used, following modifications from previous studies^29,64^. Over the course of a year, three EGB samples from each treatment were collected monthly in order to examine the EGB’s vitality over its shelf life.

### Experimental design and statistical analysis

Trials were accomplished in, at least, thrice, and the obtained data were subjected to statistical analysis. After performing a one-way ANOVA, mean averages were compared using the Tuckey test at a probability of ≤ 0.05. The statistical software: CoStat (version 6.450, CoHort Software, Birmingham, UK) was used.

## Supplementary Information

Supplementary Information 1.

Supplementary Information 2.

## Data Availability

All data generated or analyzed during this study are included within the published article and its supplementary materials. The sequencing data have been deposited in the NCBI database, with the ITS sequence under GenBank accession number ON679518. The fungal material used in this study was formally identified by Dr. Khalid M. Ghoneem Plant Pathologist and Dr. Yasser M. Shabana Plant Pathologist. A voucher specimen was not required for deposition in a publicly accessible herbarium for this study.
