# Supplementary figures and images for: Development of a granular bioformulation of Achromobacter xylosoxidans AX77 16S for sustainable onion white rot management and growth enhancement
Source: Sci Rep. 2025 Jul 30;15:27864. doi: 10.1038/s41598-025-10036-8 (PMC12311146; doi:10.1038/s41598-025-10036-8)

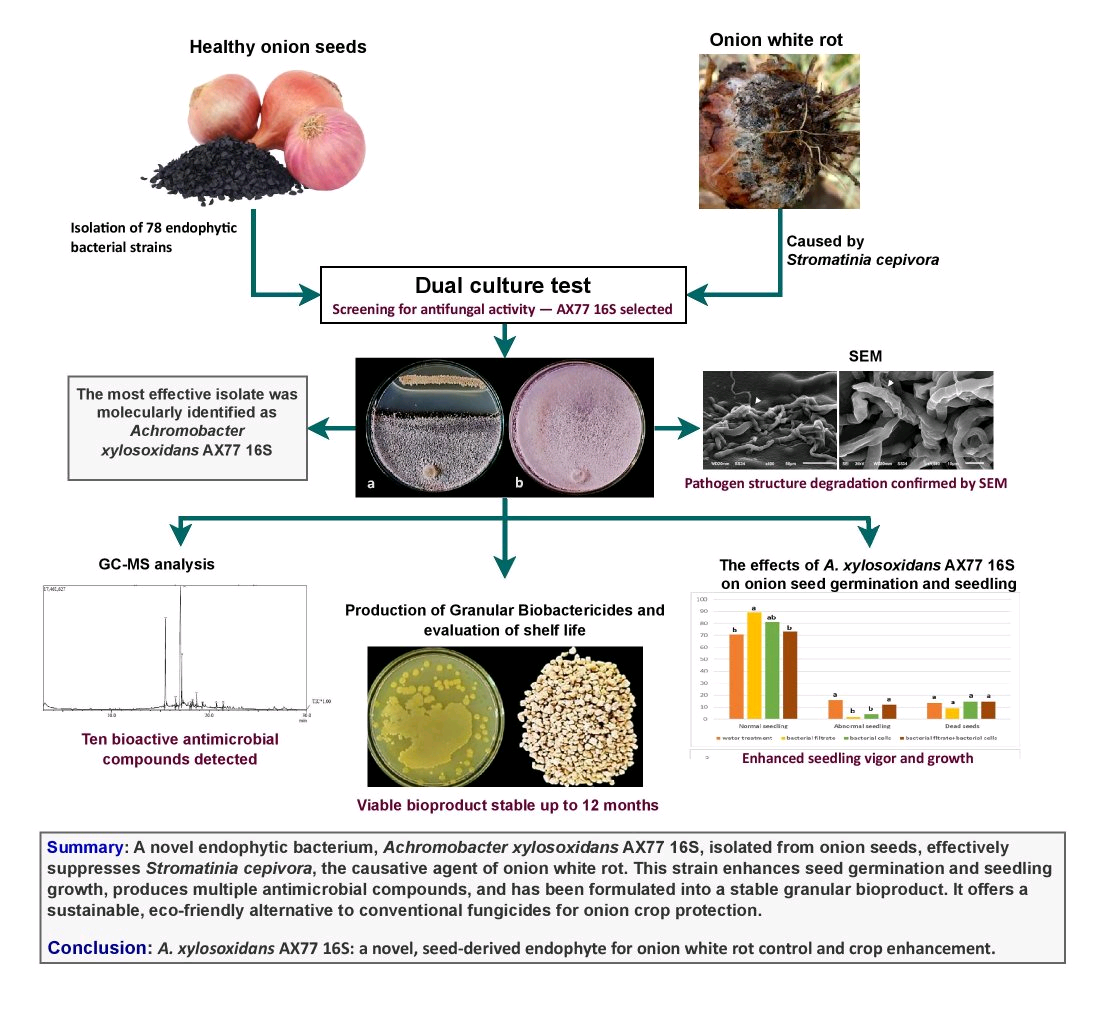

Supplement: Supplementary file 1 — Supplementary Information 1. [file 41598_2025_10036_MOESM1_ESM.jpg]
