# Supplementary material for: Development of a granular bioformulation of Achromobacter xylosoxidans AX77 16S for sustainable onion white rot management and growth enhancement
Source: Sci Rep. 2025 Jul 30;15:27864. doi: 10.1038/s41598-025-10036-8 (PMC12311146; doi:10.1038/s41598-025-10036-8)
Supplement: Supplementary file 2 — Supplementary Information 2. [file 41598_2025_10036_MOESM2_ESM.docx]

**Development of a Granular Bioformulation of *Achromobacter xylosoxidans* AX77 16S for Sustainable Onion White Rot Management and Growth Enhancement**

# Aya I. ELKasaby^1^, Khalid M. Ghoneem^2,*^, Yasser M. Shabana^1,3,*^

1. Faculty of Agriculture, Mansoura University, Mansoura 35516, Egypt; [ayaelkasaby29505@gmail.com](mailto:ayaelkasaby29505@gmail.com) (A.I.E.)

^2^Seed Pathology Research Department, Plant Pathology Research Institute, Agricultural Research Center, Giza 12619, Egypt; [khalidghoneem@arc.sci.eg](mailto:khalidghoneem@arc.sci.eg) (K.M.G.)

^3^National Council for Agricultural and Food Research, Academy of Scientific Research and Technology, Cairo 11516, Egypt; [yassershabana2@yahoo.com](mailto:yassershabana2@yahoo.com) (Y.M.S.)

***Correspondence**, [khalidghoneem@arc.sci.eg](mailto:khalidghoneem@arc.sci.eg) (K.M.G.); [yassershabana2@yahoo.com](mailto:yassershabana2@yahoo.com) (Y.M.S.)

**Table S1** The antifungal activity of endophytic bacterial isolates on the linear growth^a^ of *S. cepivora*

| **Bacterial isolate** | **Reduction (%) after** | | |
| --- | --- | --- | --- |
|  | **2 days** | **4 days** | **6 days** |
| 6MO-A 1 | 8.330 i-u**^b^** | 13.03 B-I | 34.58 m-p |
| 6MO-A 2 | 8.320 i-u | 17.60 p-D | 39.58 h-l |
| 6MO-A 4 | 8.000 i-w | 14.53 y-I | 40.00 g-l |
| 6MO-A 5 | 5.570 n-y | 14.00 z-I | 41.67 e-k |
| ARC 7 | 18.00 a-d | 22.40 d-s | 30.00 qr |
| ARC 8 | 13.87 c-j | 22.40 d-s | 34.17 n-q |
| ARC 10 | 4.440 p-y | 17.33 r-E | 28.33 r |
| ARC 12 | 4.170 r-y | 11.33 F-J | 32.08 p-r |
| ARC 13 | 6.710 k-x | 18.20 o-C | 34.58 m-p |
| 6MO-A 14 | 8.093 i-v | 12.27 D-J | 28.50 r |
| 6MO-A 18 | 5.930 l-y | 18.97 k-z | 34.17 n-q |
| 6MO-A 19 | 10.71 f-p | 23.03 c-q | 36.67 l-o |
| MO-9 21 | 2.000 v-y | 20.10 i-y | 32.50 o-r |
| MO-9 23 | 5.970 l-y | 13.33 A-I | 31.67 p-r |
| MO-9 24 | 11.90 d-m | 25.80 a-h | 38.75 j-m |
| R-A 25 | 7.150 k-x | 10.93 H-J | 16.67 s |
| R-A 27 | 4.700 p-y | 17.17 r-E | 31.25 p-r |
| R-A 28 | 10.70 f-p | 21.40 e-t | 42.08 d-j |
| R-A 29 | 10.67 f-p | 25.33 b-j | 43.75 b-h |
| R-A 30 | 9.470 g-s | 20.23 h-x | 45.47 b-e |
| R-A 32 | 17.93 a-d | 25.57 b-i | 43.75 b-h |
| ARC 33 | 17.93 a-d | 25.60 b-i | 44.17 b-g |
| ARC 35 | 8.970 g-t | 28.00 a-d | 40.47 f-l |
| ARC 36 | 7.670 j-x | 24.40 b-k | 39.17 i-l |
| AX77 | 23.00 a | 29.77 ab | 50.00 a |
| ARC 42 | 14.10 c-i | 26.20 a-f | 44.58 b-f |
| ARC 46 | 11.10 e-o | 16.30 t-H | 42.50 c-j |
| ARC 47 | 9.230 g-s | 16.33 t-H | 42.50 c-j |
| ARC 48 | 11.53 e-n | 27.40 a-d | 39.58 h-l |
| ARC 50 | 12.80 c-k | 26.20 a-f | 43.33 b-i |
| ARC 51 | 14.10 c-i | 26.80 a-e | 45.47 b-e |
| ARC 52 | 12.80 c-k | 28.60 a-c | 44.17 b-g |
| G20-A 53 | 0.000 y | 20.67 f-v | 42.50 c-j |
| G20-A 54 | 16.60 b-f | 23.00 c-q | 43.75 b-h |
| G20-A 55 | 8.530 h-t | 13.70 z-I | 37.50 k-n |
| G20-A 56 | 0.000 y | 17.77 p-D | 40.00 g-l |
| G20-A 57 | 1.730 w-y | 18.33 n-B | 41.25 e-k |
| G20-A 59 | 5.200 o-y | 17.50 q-D | 43.75 b-h |
| G20-A 61 | 5.530 n-y | 17.53 q-D | 41.67 e-k |
| G20-A 62 | 11.10 e-o | 14.50 y-I | 39.58 h-l |
| G20-A 63 | 0.000 y | 14.50 y-I | 41.25 e-k |
| G20-A 64 | 0.000 y | 19.77 j-y | 42.08 d-j |
| G20-A 65 | 0.000 y | 22.40 d-s | 42.97 c-j |
| G20-A 66 | 10.50 f-q | 12.70 C-I | 45.00 b-e |
| G20-B 69 | 8.730 h-t | 20.03 i-y | 42.50 c-j |
| G20-B 70 | 7.000 k-x | 20.60 f-v | 42.08 d-j |
| G20-B 71 | 0.000 y | 18.77 l-A | 42.50 c-j |
| G20-B 72 | 16.60 b-f | 15.13 v-I | 43.75 b-h |
| G20-B 73 | 5.530 n-y | 15.70 u-I | 39.17 i-l |
| G20-B 74 | 11.07 e-o | 11.07 G-J | 38.75 j-m |
| R-B 75 | 7.000 k-x | 31.33 a | 40.00 g-l |
| R-B 76 | 23.00 a | 24.03 c-m | 40.47 f-l |
| R-B 77 | 20.43 ab | 12.30 D-J | 33.75 n-q |
| 6MO-B 78 | 9.770 g-r | 17.27 r-E | 30.00 qr |
| 6MO-B 79 | 12.67 c-k | 1.83 KL | 31.25 p-r |
| 6MO-B 80 | 7.430 k-x | 15.40 u-I | 30.47 p-r |
| 6MO-B 81 | 5.100 o-y | 20.33 g-w | 37.50 k-n |
| 6MO-B 82 | 12.53 d-k | 25.27 b-j | 33.75 n-q |
| GR 83 | 20.43 ab | 25.87 a-g | 33.75 n-q |
| GR 84 | 23.00 a | 14.77 w-I | 36.67 l-o |
| GR 85 | 9.770 g-r | 0.000 L | 42.08 d-j |
| GR 87 | 15.10 b-g | 0.000 L | 42.50 c-j |
| NG 91 | 1.430 xy | 18.13 o-C | 45.00 b-e |
| NG 92 | 7.370 k-x | 21.43 e-t | 42.50 c-j |
| NG 93 | 2.200 u-y | 24.67 b-j | 41.60 e-k |
| NG 94 | 0.000 y | 14.07 z-I | 45.47 b-e |
| ARC 97 | 12.90 c-k | 13.90 z-I | 30.00 qr |
| NG 98 | 7.400 k-x | 23.87 c-n | 40.47 f-l |
| NG 103 | 8.730 h-t | 24.80 b-j | 44.58 b-f |
| NG 104 | 7.400 k-x | 15.70 u-I | 34.17 n-q |
| NG 105 | 7.400 k-x | 0.000 L | 42.08 d-j |
| NG 108 | 0.000 y | 22.40 d-s | 45.00 b-e |
| NG 111 | 8.630 h-t | 10.10 IJ | 13.75 s |
| NG 112 | 7.200 k-x | 13.90 z-I | 33.75 n-q |
| NG 113 | 0.000 y | 10.90 H-J | 40.00 g-l |
| GW 114 | 0.000 y | 16.93 s-F | 38.75 j-m |
| T 117 | 1.430 xy | 11.80 E-J | 39.58 h-l |
| T 121 | 8.670 h-t | 4.470 KL | 37.50 k-n |
| P value at 0.05 | <0.0001 | <0.0001 | <0.0001 |

^a^ Values are the average of four replicates.

^b^ Within each column, values sharing a letter are not significantly different according to Tukey's HSD test (*P* ≤ 0.05) (n=4 plates/ bacterial isolate).
